# Posterior lamellar versus bilamellar tarsal rotation surgery for trachomatous trichiasis in Ethiopia: a randomised controlled trial

**DOI:** 10.1016/S2214-109X(15)00299-5

**Published:** 2016-01-14

**Authors:** Esmael Habtamu, Tariku Wondie, Sintayehu Aweke, Zerihun Tadesse, Mulat Zerihun, Zebideru Zewudie, Amir Bedri Kello, Chrissy H Roberts, Paul M Emerson, Robin L Bailey, David C W Mabey, Saul N Rajak, Kelly Callahan, Helen A Weiss, Matthew J Burton

**Affiliations:** a London School of Hygiene & Tropical Medicine, London, UK; b The Carter Center, Addis Ababa, Ethiopia; c Amhara Regional Health Bureau, Bahirdar, Ethiopia; d Light for the World, Addis Ababa, Ethiopia; e International Trachoma Initiative, Atlanta, GA, USA; f The Carter Center, Atlanta, GA, USA

## Abstract

**Background:**

Eyelid surgery is done to correct trachomatous trichiasis to prevent blindness. However, recurrent trichiasis is frequent. Two procedures are recommended by WHO and are in routine practice: bilamellar tarsal rotation (BLTR) and posterior lamellar tarsal rotation (PLTR). This study was done to identify which procedure gives the better results.

**Methods:**

A randomised, controlled, single masked clinical trial was done in Ethiopia. Participants had upper lid trachomatous trichiasis with one or more eyelashes touching the eye or evidence of epilation, in association with tarsal conjunctival scarring. Exclusion criteria were age less than 18 years, recurrent trichiasis after previous surgery, hypertension, and pregnancy. Participants were randomly assigned (1:1) to either BLTR or PLTR surgery, stratified by surgeon. The sequences were computer-generated by an independent statistician. Surgery was done in a community setting following WHO guidelines. Participants were examined at 6 months and 12 months by assessors masked to allocation. The primary outcome was the cumulative proportion of individuals who developed recurrent trichiasis by 12 months. Primary analyses were by modified intention to treat. The intervention effect was estimated by logistic regression, controlled for surgeon as a fixed effect in the model. The trial is registered with the Pan African Clinical Trials Registry (number PACTR201401000743135).

**Findings:**

1000 participants with trichiasis were recruited, randomly assigned, and treated (501 in the BLTR group and 499 in the PLTR group) between Feb 13, 2014, and May 31, 2014. Eight participants were not seen at either 6 month or 12 month follow-up visits and were excluded from the analysis: three from the PLTR group and five from the BLTR group. The follow-up rate at 12 months was 98%. Cumulative recurrent trichiasis by 12 months was more frequent in the BLTR group than in the PLTR group (110/496 [22%] *vs* 63/496 [13%]; adjusted odds ratio [OR] 1·96 [95% CI 1·40–2·75]; p=0·0001), with a risk difference of 9·50% (95% CI 4·79–14·16).

**Interpretation:**

PLTR surgery was superior to BLTR surgery for management of trachomatous trichiasis, and could be the preferred procedure for the programmatic management of trachomatous trichiasis.

**Funding:**

The Wellcome Trust.

## Introduction

Trachoma, a neglected tropical disease caused by *Chlamydia trachomatis*, is the leading infectious cause of blindness.^1^ Recurrent infection drives progressive conjunctival scarring, which turns the lid and eyelashes in towards the eye (trichiasis) resulting in pain and eventually blinding corneal opacification. About 1·2 million people are irreversibly blind from this disease and about 7·2 million have trichiasis.^1^, ^2^ WHO recommends the SAFE strategy for trachoma control: Surgery for trichiasis, Antibiotics, Facial cleanliness, and Environmental improvement.^3^ Trichiasis surgery reduces the risk of sight loss by correcting the in-turned eyelid, thus stopping the corneal damage. Surgery involves an incision through the scarred upper eyelid, parallel to the lid margin, outward rotation, and suturing in the corrected position.^4^ Due to the limited number of ophthalmologists in most trachoma-endemic countries, surgery is usually done by non-physicians with limited training, equipment, and time.^3^ Given these constraints, the technique needs to be simple, safe, and quick to do, whereas at the same time giving consistently good results.

Unfortunately, trichiasis frequently recurs after surgery. This outcome represents a substantial limitation in preventing sight loss from trachoma. Studies have reported trichiasis recurrence rates between 10% at 3 months and up to 60% at 3 years, with an average of around 20% at 1 year.^5^, ^6^, ^7^, ^8^, ^9^, ^10^, ^11^, ^12^, ^13^, ^14^ Several factors contribute to recurrent trichiasis, including preoperative disease severity, surgeon skill, and surgical procedure.^15^ Among these, operation type is a major determinant of outcome and subtle variations in procedure performance probably affect results.^10^, ^11^, ^16^ Many different surgical procedures have been used to correct trichiasis, with some evidence that bilamellar tarsal rotation (BLTR) is better than others to which it has been formally compared.^10^, ^11^, ^15^, ^17^ However, it is important to determine which is the best of these options.

**Research in context**

**Evidence before this study**

Members of our study group recently published a systematic review of the management of trachomatous trichiasis (Burton and colleagues, 2015). When preparing this systematic review, we searched CENTRAL, Ovid MEDLINE, Embase, ISRCTN registry, ClinicalTrials.gov, and WHO ICTRP. We searched until May 7, 2015, using the search terms “trachoma” and “trichiasis”. See the review's appendix for full search methods for each database. We identified one previous randomised trial (Adamu and Alemayehu, 2002), which compared variants of the BLTR and PLTR procedure done by ophthalmologists in a teaching hospital environment in Ethiopia; 153 patients were randomly assigned and followed for 3 months. No evidence of a difference in outcome was found. However, this earlier study was constrained by a small sample size and short duration. The surgery was performed in a teaching hospital setting by ophthalmologists, in contrast to the health centre provision by non-physicians typical of trachoma control programmes, limiting the conclusions that can been drawn.

**Added value of this study**

Our trial was designed to compare the two most common operations used to treat trachomatous trichiasis to determine which gives the best results in terms of disease recurrence and complications in a programmatic setting. The results show that the PLTR was superior to BLTR because it had a substantially lower trichiasis recurrence rate by 1 year and fewer intraoperative and immediate postoperative complications.

**Implications of all the available evidence**

This study provides evidence of superiority of PLTR, suggesting that it could be the best procedure for the programmatic management of trachomatous trichiasis. We suggest that new surgical trainees in both established and new programmes be trained in the PLTR procedure. Another trial examining the outcomes of PLTR surgery done by surgeons previously trained in BLTR surgery should be considered.

About 20 years ago several procedures were compared with the BLTR operation in randomised controlled trials.^10^, ^11^ The findings from these trials showed that the BLTR procedure had the lowest trichiasis recurrence rate of the procedures compared (about 20% at 1 year), leading WHO to recommend it as the preferred operation.^3^ However, the most commonly used alternative procedure, the posterior lamellar tarsal rotation (PLTR) or Trabut operation, was not included in these earlier trials. One earlier randomised trial from Ethiopia compared variants of the BLTR and PLTR, and found no difference. However, that trial was relatively small, with only 3 months' follow-up and was done by ophthalmologists in a teaching hospital, precluding conclusions for control programmes that do the vast majority of trichiasis surgery.^13^

There is an unprecedented effort to scale up global trichiasis surgery output and improve outcomes, to clear the huge trichiasis backlog. This effort requires training many trichiasis surgeons on the easiest, safest, and most successful operation with the least recurrence and complications. There is an urgent need to examine rigorously which of these two most frequently performed operations has the best outcomes in a programmatic setting, with an adequate sample size and follow-up period. This question was identified as a research priority several years ago by the WHO Alliance for the Global Elimination of Trachoma by 2020 (GET2020).^18^ The aim of our trial was to determine whether BLTR or PLTR surgery gives superior results under programmatic conditions.

## Methods

### Study design and participants

This was a single-masked, individual-randomised, controlled trial done in Ethiopia. Adults with trachomatous trichiasis were randomly allocated to either BLTR or PLTR surgery, stratified by surgeon, and followed up for 1 year. The study was approved by the Ethiopian National Health Research Ethics Review Committee, the London School of Hygiene & Tropical Medicine Ethics Committee, Emory University Institutional Review Board, and the Ethiopia Food, Medicine and Healthcare Administration and Controls Authority. The trial was done in compliance with the Declaration of Helsinki and International Conference on Harmonisation–Good Clinical Practice. An independent data and safety monitoring committee oversaw the trial.

Participants had upper lid trachomatous trichiasis with one or more eyelashes touching the eye or evidence of epilation, in association with tarsal conjunctival scarring. We excluded people with trichiasis due to other causes, recurrent trichiasis after previous surgery, hypertension, pregnancy, and those under 18 years. Patients were recruited mainly through community-based screening in three districts of West Gojam Zone, Amhara Region, Ethiopia. Recruitment and surgery were performed in community level health centres. Written informed consent in Amharic was obtained before enrolment from participants. If a participant was unable to read and write, the information sheet and consent form were read to them and their consent recorded by thumbprint.

### Randomisation and masking

Participants were randomly assigned (1:1) to either PLTR or BLTR surgery for each surgeon, with random block sizes of 4 or 6. Randomisation was stratified by surgeon because of potential intersurgeon variability. The sequences were computer-generated by an independent statistician. Separate allocation sequences for each surgeon were concealed in sequentially numbered, sealed, opaque envelopes. A person independent of all other aspects of the trial prepared these envelopes.

On most recruitment days, two surgeons operated simultaneously. Following baseline examination, participants were allocated to the next available surgeon. A fieldworker was responsible for implementing the intervention assignment in a dedicated area. The fieldworker and surgeon jointly confirmed the allocation and recorded this in the surgical logbook. The different surgical equipment sets for the two procedures were kept separately. The randomisation fieldworker and surgeon jointly collected the appropriate surgical set for the allocated procedure. Surgeons and patients were aware of the allocation. The two examiners (EH, SA) who were responsible for clinical observations at baseline, 6 months, and 12 months were masked to the allocation. They were not involved in the allocation process, surgery, postoperative care, or the 10 day follow-up. The intraoperative and 10 day observations were made by separate fieldworkers who could not be masked to the allocation.

### Procedures

At the preoperative assessment before randomisation, demographic characteristics were recorded. Presenting logMAR (logarithm of the minimum angle of resolution) visual acuity at 2 m was measured using PeekAcuity software on a smartphone in a dark room.^19^ For visual acuities of counting fingers or less, logMAR values were attributed as follows: counting fingers, 2·0; hand movements, 2·5; perception of light, 3·0; and no perception of light, 3·5.^8^ We assessed contrast sensitivity with a prototype smartphone-based test that presents calibrated grey scale spots against a white background, which are identified by touch.

Eyes were examined by a single examiner (EH) using 2·5× binocular loupes and torch, and graded using the Detailed WHO FPC Grading System.^20^ Lashes touching the eye were counted and subdivided by the part of the eye contacted: cornea, lateral, or medial conjunctiva. Trichiasis subtypes were recorded: metaplastic, misdirected, and entropic.^21^ Clinical evidence of epilation was identified by broken or newly growing lashes, or areas of absent lashes. Upper lid entropion was graded by assessing the degree of eyelid margin inward rotation.^21^ Corneal scarring was graded using a previously described detailed system.^20^ Three standardised high-resolution digital photographs of trichiasis, cornea, and tarsal conjunctiva were taken, using a Nikon D90 digital SLR camera with 105 mm macro lens and R1C1 flash units.^22^

Before recruitment, nine experienced trichiasis nurse-surgeons, already trained, certified, and regularly performing PLTR surgery were trained in BLTR surgery. We followed the procedures described in the WHO Trichiasis Surgery for Trachoma manual.^4^ After training, surgeons were carefully observed throughout five operations and certified as correctly doing the procedure following the standardisation checklist.^4^ Surgeons then returned to their usual workplace, and regularly performed BLTR for 6 months. They then returned for repeat standardisation, assessment, and certification on both PLTR and BLTR procedures by two assessors. Before commencing the trial, each surgeon had done about 100 BLTR procedures (median 117, range 94–137). The best six surgeons did the surgery in this trial: they were all certified as consistently performing all component steps of both operations correctly, using the WHO certification procedures.

The procedures are described in detail in the WHO manual.^4^ Briefly, in the PLTR the eyelid is everted, an incision is made through the tarsal conjunctiva and tarsal plate (posterior lamella), parallel to and 3 mm above the lid margin. The posterior lamella is separated from the anterior lamella (orbicularis muscle and skin). Three sutures are placed to externally rotate and fix the eyelid. In the BLTR the eyelid is fixed with a clamp (Waddell type), of an appropriate size.^23^ A full-thickness incision is made through the anterior and posterior lamellae, parallel to and 3 mm above the lid margin. Three sutures are placed to externally rotate and fix the eyelid. Surgery was done under subcutaneous local anaesthesia (2–3 mL of lidocaine 2%, with adrenaline). In both surgical procedures, 4/0 silk sutures with 3/8th circle, 19 mm cutting needles were used. Surgery duration was measured and complications documented. Postoperatively, operated eyes were padded for 1 day and tetracycline eye ointment 1% was self-administered twice daily for 2 weeks. Participants were not given perioperative oral azithromycin because it is not the operational practice to use it in this region.

Participants were examined at 10 days, 6 months, and 12 months after operation. At 10 days, data were collected on patient-reported outcomes (improvement in preoperative symptoms, postoperative pain, and functioning). Participants were examined for recurrence, degree of lid eversion, infection, granulomata, and eyelid contour abnormality (ECA) before suture removal.

At 6 months and 12 months participants were re-examined following the same procedures as for baseline (SA at 6 months and EH at 12 months). The examiners were standardised and had very strong agreement for the primary outcome in grading validation studies (κ=0·95). Based on severity, trichiasis cases were categorised into minor trichiasis with less than six lashes or evidence of epilation in less than one third of the lid margin, and major trichiasis with six or more lashes or evidence of epilation in one third or more of the lid margin. The degree of entropion correction was graded as follows: (grade 1) extra eversion: main lashes point superiorly, whole lid margin visible, and tarsal plate surface visible; (grade 2) lid margin eversion: lashes point superiorly, whole lid margin visible, and tarsal surface not visible; (grade 3) partial lid margin entropion: some parts of the lashes might point anteriorly and some part of the lid margin not visible; (grade 4) total lid margin entropion: lashes might point inferiorly or towards the globe and lid margin is not visible. We considered grade 1 over-correction, grade 2 normal correction, and grades 3 and 4 under-correction. Granulomata were defined as fleshy tissue growth of at least 2 mm on the tarsal conjunctiva or at the edge of the eyelid.^12^ Grading of ECAs was based on the PRET trial method: mild, vertical deviation from the natural contour less than 1 mm in height and affecting more than one third of horizontal eyelid length; moderate, vertical deviation from the natural contour 1–2 mm in height or affecting one third to two thirds of horizontal eyelid length; severe, vertical deviation from the natural contour more than 2 mm in height or a defect more than two thirds the horizontal eyelid length.^24^ These were regrouped as: clinically non-significant ECA, which included mild ECA; and clinically significant ECA, which included moderate-to-severe ECA. The clinically significant ECAs also included other ECAs such as divot, which is a scarred depression or tissue loss including lashes at the eyelid margin. Visual acuity and contrast sensitivity were measured at 12 months. Data on patient-reported outcomes were collected at 12 months. Individuals with recurrent trichiasis during follow-up were offered repeat surgery. Participants with other ophthalmic pathology (eg, cataract) were referred.

High-resolution digital photographs of upper eyelid, cornea, and tarsal conjunctiva were taken at 6 months and 12 months.^22^ To address potential concerns of bias which might arise from identifying procedure type from surgical scars, the upper eyelid photograph was taken after covering the incision area with a shaped occluder to prevent any unmasking of the independent photograph grader (an ophthalmologist with 15 years' experience of examining for trachomatous trichiasis). Images were viewed on a 15 inch high-resolution “retina” screen (Apple). Trachomatous trichiasis was considered to be present if there was one or more lashes touching the eye, identified by the lashes deviating over the globe and appearing to touch the eye.

### Outcomes

The primary outcome was the cumulative proportion of individuals who developed recurrent trichiasis by 12 months. Recurrent trichiasis was defined as one or more lashes touching the eye or clinical evidence of epilation, or a history of repeat trichiasis surgery by 12 months. A-priori defined secondary outcome measures were: recurrent trichiasis at 6 months and 12 months; trichiasis recurrence difference by surgeon; trichiasis recurrence difference by baseline disease severity; number, type, and location of recurrent lashes at 12 months; corneal opacity, vision, and contrast sensitivity changes at 12 months; intraoperative, immediate, and late postoperative surgical complications (bleeding, infection, and granulomas); ECA at 12 months; and patient-reported outcomes.

### Statistical analysis

In the STAR trial, the 1 year trichiasis recurrence rate using BLTR surgery was about 10% (tetracycline group).^5^ In our recent trials in Ethiopia involving patients with a similar severity of disease to the STAR trial, we found PLTR surgery had a 1 year recurrence rate of 18%.^8^ A sample of 836 participants was estimated to have 90% power and 95% confidence to detect a similar difference in recurrent trichiasis (18% *vs* 10%). Therefore, we aimed to recruit 1000 cases (500 in each group), to allow for about 15% loss to follow-up.

Data were double-entered into Access 13 (Microsoft) and transferred to Stata 11 (StataCorp) for analysis. For participants who had bilateral surgery, we randomly designated one eye to be the study eye for the analysis. A modified intention-to-treat analysis was done, with primary outcome data analysed on all participants seen at either the 6 month or 12 month follow-up or both. Those not seen at either of these follow-up visits were excluded from the analysis.

The primary outcome and binary secondary outcomes were compared between the two surgical groups with logistic regression analyses to estimate the odds ratio (OR) and 95% CI. All comparisons between the two surgical procedures were controlled for surgeon as a fixed effect in the model to account for the stratified randomisation. The risk difference in the primary outcome (recurrent trichiasis by 12 months) between BLTR and PLTR procedures was estimated. The possibility of effect modification between group and a-priori defined factors such as surgeon, preoperative trichiasis severity, papillary inflammation, age, and sex was investigated by including interaction terms in the model and using a likelihood ratio test to assess statistical significance of the interaction term. Ordered categorical secondary outcomes (changes in visual acuity and corneal opacity, bleeding, and patient-reported outcomes) were compared between the two surgical interventions using ordinal logistic regression. Categorical secondary outcomes (type and location of recurrent lashes, ECAs, and entropion correction) were analysed using multinomial logistic regression to estimate relative risk ratio (RRR) and 95% CI. Negative binomial regression was used to analyse the difference in the number of recurrent lashes touching the eye between the two intervention groups. The signed-rank test was used to analyse visual acuity and contrast sensitivity changes between baseline and the 12 month follow-up. The risk of trichiasis recurrence difference by surgeon between the two surgical interventions was analysed using logistic regression adjusted for baseline disease severity such as entropion and trichiasis. To investigate the possibility of a learning curve effect during recruitment, the trichiasis recurrence rates for the first 50% of cases to be recruited versus the second 50% of cases recruited for each surgeon were compared using logistic regression adjusted for baseline disease severity such as entropion and trichiasis. The trial is registered at the Pan African Clinical Trials Registry (PACTR201401000743135).

### Role of the funding source

The funder of the study had no role in study design, data collection, data analysis, data interpretation, or writing of the report. The corresponding author had full access to all the data in the study and had final responsibility for the decision to submit for publication.

## Results

Between Feb 13, 2014, and May 31, 2014, 5168 people were examined for eligibility, of whom 1314 (25%) had trachomatous trichiasis (figure). The remaining 3854 had other ocular conditions. Of the 1314 trichiasis cases, 312 did not meet inclusion criteria, largely because they had previously received surgery for trichiasis. Of the 1002 eligible participants, two (<1%) declined surgery. Thus, 1000 trichiasis cases consented, were enrolled, and randomly assigned (501 in the BLTR group and 499 in the PLTR group).

Participants were reassessed at 10 days (range 7–14) for suture removal, 6 months, and 12 months after enrolment. Almost all (98%) participants were examined at each follow-up. At 10 days, two people had travelled to another region, and had sutures removed in their new locality. Eight (1%) participants were not seen at either 6 month or 12 month follow-up visits and were therefore excluded from the analysis: three from the PLTR group and five from the BLTR group. Hence, primary outcome data were available and analysed for 992 (99%): 496 in each group.

Baseline demographic and clinical characteristics were balanced between the trial groups (table 1). The majority of the participants were female (77%) and their mean age was 47·3 years. The two groups were comparable for visual acuity and prevalence of corneal opacity, conjunctival inflammation, scarring, entropion, and trichiasis. There was evidence of epilation in 588 (59%) participants: 281 (56%) in the PLTR group and 307 (61%) in the BLTR group; among these 82 (8%) in both groups had successfully epilated, with no lashes touching. Major trichiasis was present in 145 (29%) of the PLTR group and 144 (29%) of the BLTR group. About 90% of the participants in both groups had corneal lashes (table 1). PLTR surgery took slightly less time than BLTR surgery (15 min 33 s *vs* 16 min 39 s; p<0·0001).

By 12 months, the primary outcome, cumulative recurrent trichiasis, had developed in 173 (17%) of 992 study eyes. Cumulative recurrence was significantly more frequent in the BLTR group (110/496 [22%]) than in the PLTR group (63/496 [13%]); after adjusting for surgeon, the OR was 1·96 (95% CI 1·40–2·75; p=0·0001). The risk difference for recurrent trichiasis between BLTR and PLTR procedures was 9·50% (95% CI 4·79–14·16). There was no evidence of effect modification between group and a-priori defined other factors on the primary outcome, including surgeon.

The primary outcome analysis using the photograph grading results was similar to the field grading. By 12 months, cumulative recurrent trichiasis was recorded for 250 (25%) of 992 study eyes. Recurrence was significantly more frequent in the BLTR group than in the PLTR group (32% *vs* 19%; OR 1·97 [95% CI 1·47–2·65]; p<0·0001). The risk difference for recurrent trichiasis between BLTR and PLTR procedures was 12·5% (95% CI 7·2–17·8).

At 10 days, recurrent trichiasis was present in three study eyes, one in the PLTR group and two in the BLTR group. At 6 months, recurrent trichiasis was present in 114 (12%) of 983 study eyes, and was significantly more frequent in the BLTR group than the PLTR group (71 [14%] *vs* 43 [9%]; OR 1·77 [95% CI 1·19–2·65]; p=0·0001). At 12 months, recurrent trichiasis was present in 131 (13%) of 981 study eyes and again remained significantly more frequent in the BLTR group than the PLTR group (85 [17%] *vs* 46 [9%]; OR 2·04 [95% CI 1·39–2·99]; p=0·0003).

There was no evidence of a difference in the risk of trichiasis recurrence between surgeons by 12 months for either PLTR (p=0·80) or BLTR (p=0·44), or for a learning curve during the course of the trial for either procedure. For PLTR, recurrence risks during the first and second half of recruitment were 32 (13%) of 248 and 31 (12%) of 248, respectively (p=0·68). For BLTR, recurrence risks during the first and second half of recruitment were 55 (22%) of 247 and 55 (22%) of 249, respectively (p=0·93).

The number, type, and location of recurrent lashes were comparable between the two groups (Table 1, Table 2). BLTR surgery had more frequent recurrence than PLTR surgery for major trichiasis cases and across all baseline entropion grades. There was no evidence of a difference in visual acuity, contrast sensitivity, corneal opacity, and entropion changes at 12 months between the two groups (table 2). However, compared with the baseline, at 12 months there was a statistically significant overall improvement in visual acuity (baseline median logMAR, 0·6 [IQR 0·3–0·8] *vs* 12 month median logMAR, 0·5 [0·2–0·7]; signed-rank test, p<0·0001) and contrast sensitivity (baseline median contrast sensitivity, 3% [2–5] *vs* 12 month contrast sensitivity, 2% [1–3]; signed-rank test, p<0·0001) in the entire combined study sample.

After adjusting for surgeon, there was evidence of a difference in odds of intraoperative, immediate, or late postoperative complications between the two surgical interventions (table 3). There was more intraoperative and immediate postoperative bleeding in the BLTR surgery group than the PLTR surgery group (OR 2·76 [95% CI 1·27–6·00]; p=0·01), and also more postoperative infection in the BLTR surgery group than the PLTR surgery group (OR 4·44 [95% CI 2·11–9·33]; p=0·0001; table 3). Granulomata were less frequent in the BLTR group compared with the PLTR group (OR 0·41 [95% CI 0·20–0·83]; p=0·01; table 3).

The frequency of clinically non-significant (mild) ECA at 12 months was lower in the BLTR surgery group than the PLTR surgery group (RRR 0·50 [95% CI 0·34–0·73]; p<0·0001; table 3). However, there was no evidence of a difference in the frequency of clinically significant (moderate-to-severe) ECA between the two groups (RRR 1·10 [95% CI 0·66–1·81]; p=0·72; table 3). A similar pattern in ECA was found by independent photograph grading. Clinically mild ECA at 12 months was less frequent in the BLTR group (27/484 [5%]) than in the PLTR group (58/489 [12%]; RRR 0·43 [95% CI 0·27–0·70]; p=0·001). However, again we found no evidence of a difference in moderate-to-severe ECA between the two groups (BLTR, 4% *vs* PLTR, 5%; RRR 0·76 [95% CI 0·41–1·44]; p=0·40). There was evidence of more under-correction at 12 months with BLTR surgery than PLTR surgery (table 3).

There was no evidence of a difference between groups in the patient-reported pain experienced during surgery (p=0·84; table 4). However, participants in the BLTR group reported more pain and discomfort during the days between surgery and suture removal than the PLTR group (OR 1·46 [95% CI 1·12–1·89]; p=0·004; table 4). There was no evidence of a difference in patient satisfaction between the two groups for treatment of trichiasis (p=0·20) or the cosmetic appearance of the operated eyelid (p=0·64; table 4).

## Discussion

Around 7 million people have trachomatous trichiasis and require high-quality surgical intervention.^2^ A major global effort exists to scale up surgical programmes. However, high postoperative trichiasis recurrence rates are undermining trachoma control.^25^ Identifying the surgical intervention with the lowest recurrence rate has been a research priority for many years.^18^ In this trial, we compared the relative effectiveness of the two most commonly used operations and found that PLTR surgery has a significantly lower trichiasis recurrence rate at 12 months than BLTR surgery, particularly for more severe cases.

Considerable care was taken to ensure that the surgeons did both procedures using the WHO-described method with equal precision.^4^ We trained surgeons who had been previously taught PLTR to do the BLTR procedure. This approach was chosen, rather than training novice surgeons simultaneously in both procedures, to reduce the learning curve to achieve proficiency in the new procedure.^26^ During training and standardisation, before the commencement of the trial, each surgeon did about 100 BLTR operations and was confirmed by two assessors to be performing the procedure per protocol, using the WHO Certification process.^4^

There is clear evidence that during the trial the surgeons continued to do both operations consistently well and that the recorded difference in the primary outcome was not attributable to having learnt the BLTR procedure more recently. First, there were only three recurrent cases by 10 days, indicating that primary surgical failure was rare. If the recorded differences in recurrence were due to poor surgical technique, we would anticipate this to be more apparent by 10 days. This finding suggests that the subsequent difference in the primary outcome is attributable to fundamental differences in the surgical method that achieves a more stable and long-lasting correction in the case of PLTR surgery. Second, trichiasis recurrence rates between the first and second half of recruitment were very similar. If surgeons were still on a BLTR learning curve, a lower recurrence rate in the second half of recruitment would have been anticipated. Third, there was no significant difference in recurrence for either surgical procedure between surgeons. Finally, the recurrence rates for both procedures were generally similar to or lower than those reported in other trials, with the exception of the STAR trial which reported a lower BLTR recurrence rate.^5^, ^6^, ^7^, ^8^, ^9^, ^10^, ^11^, ^12^, ^13^, ^14^

The only other trial to compare BLTR and PLTR procedures was done in Ethiopia.^13^ This trial reported comparable outcomes for the two procedures: BLTR, 10·4%, and PLTR, 12·3%, recurrence at 3 months. However, this earlier study had a number of constraints. First, it was under-powered to detect a difference (153 patients, 256 eyes operated). Second, it was done at a tertiary teaching hospital by ophthalmologists. By contrast, most programmatic trichiasis surgery is done by non-physicians with limited training in remote, low-level, health facilities. Alternative techniques might give different results in more programmatic settings. Third, the 3 month follow-up period was too short to assess the relative performance because differences might take longer to become apparent.^6^, ^7^, ^11^, ^27^, ^28^

The PLTR surgical procedure did better than BLTR for several secondary outcomes. A higher rate of postoperative infection occurred following BLTR, probably because of the skin incision. All infections were treated successfully with oral antibiotics. The skin and orbicularis incision probably also explains the greater intraoperative and postoperative bleeding and postoperative pain that occurred with the BLTR procedure because these structures have an extensive vascular and sensory supply. These are important considerations for improving surgical uptake, which might be reduced by patient reports of pain and bleeding. Participants reported very high levels of satisfaction with the cosmetic outcome and effect of surgery in alleviating the trichiasis in both groups at 12 months. There was no difference by group. However, some caution needs to be taken in drawing firm conclusions from such data; the questions were asked by members of the study team, and there could be some reticence in expressing dissatisfaction in this context. Of note, the Kenyan National Trachoma Control Programme recently switched from the BLTR to PLTR surgery because of reports of widespread patient dissatisfaction with the appearance of the full-thickness incision in BLTR surgery. Under-correction was more frequent with BLTR surgery at 12 months, suggesting it is less effective at correcting underlying entropion.

We found that BLTR had a lower rate of mild ECAs. We consider this degree of ECA to be clinically and cosmetically non-significant because the vertical deviation from the lid contour is less than 1 mm. It is possible that this difference reflects consistently greater degrees of evertion with PLTR. There was no difference in moderate-to-severe ECA by group. Conjunctival granulomata developed more frequently after PLTR surgery.^8^ Granulomata are probably a vigorous healing response that occurs in a tissue defect.^12^ The additional rotation effected by the PLTR might create a larger posterior lamella defect and thereby a higher likelihood of granuloma formation. However, they are usually a minor complication that either self-resolve or need only a simple shave under topical anaesthesia. In the earlier comparison of BLTR and PLTR in Ethiopia, both eyelid notching and granulomata were significantly more common in the BLTR group than in the PLTR group (p=0·002).^13^

We think that it is biologically plausible that the PLTR achieves a more effective and stable correction of the entropion and trichiasis due to a key difference in technique from the BLTR. In the PLTR procedure, the lower edge of the dissected upper portion of the tarsal plate is drawn down and tucked into the dissected space between the anterior lamella and the lower portion of the tarsal plate behind.^4^ Once healed, this provides a wedge of tissue that continues to rotate the distal end of the eyelid outwards, and stabilise the correction.

This study has several strengths. It had a large sample size and very high follow-up rates. Demographic and clinical characteristics were balanced between groups. The surgeons were rigorously trained and standardised to ensure the procedures were done correctly.

A potential design limitation in a trial of these two procedures is the risk of unmasking at the time of follow-up observations because some BLTR cases might very occasionally have a faint skin scar. The baseline, 6 month, and 12 month observers were masked to the randomisation. However, to independently assess the primary outcome for observer bias, photographs in which the upper lid skin was covered by a mask were graded. We found that the analysis of primary outcome using field and photograph grading were comparable, showing no systematic bias in the field grading. The observations of some of the secondary outcomes made during the operative procedure and at 10 days were impossible to mask. The use of surgeons who had previously been trained in PLTR and then provided with a second round of training in BLTR could be viewed as a potential limitation. However, we think that there was ample pre-trial training, practice, and assessment to bring the surgeons to a proficient standard, and that there is clear evidence that during the trial high standards were maintained, as discussed above. In this trial we used silk sutures, which were removed at 10 days. Although absorbable sutures such as polyglactan-910 (vicryl) offer the operational advantage of not needing to be removed, we have previously found in a randomised trial that silk and absorbable sutures have comparable outcomes, and therefore it is unlikely that the outcome of this present study would be modified by their use.^8^

Overall, the PLTR procedure was superior to the BLTR in terms of lower trichiasis recurrence and fewer intraoperative and immediate postoperative complications. All other factors being equal, PLTR could be the preferred procedure for the programmatic management of trachomatous trichiasis. We suggest new surgical trainees in both established and new programmes should be trained in the PLTR procedure. Additionally, consideration could be given to further research to investigate whether individuals previously trained to do BLTR surgery need to be re-trained in PLTR surgery.

## Figures and Tables

**Figure:**
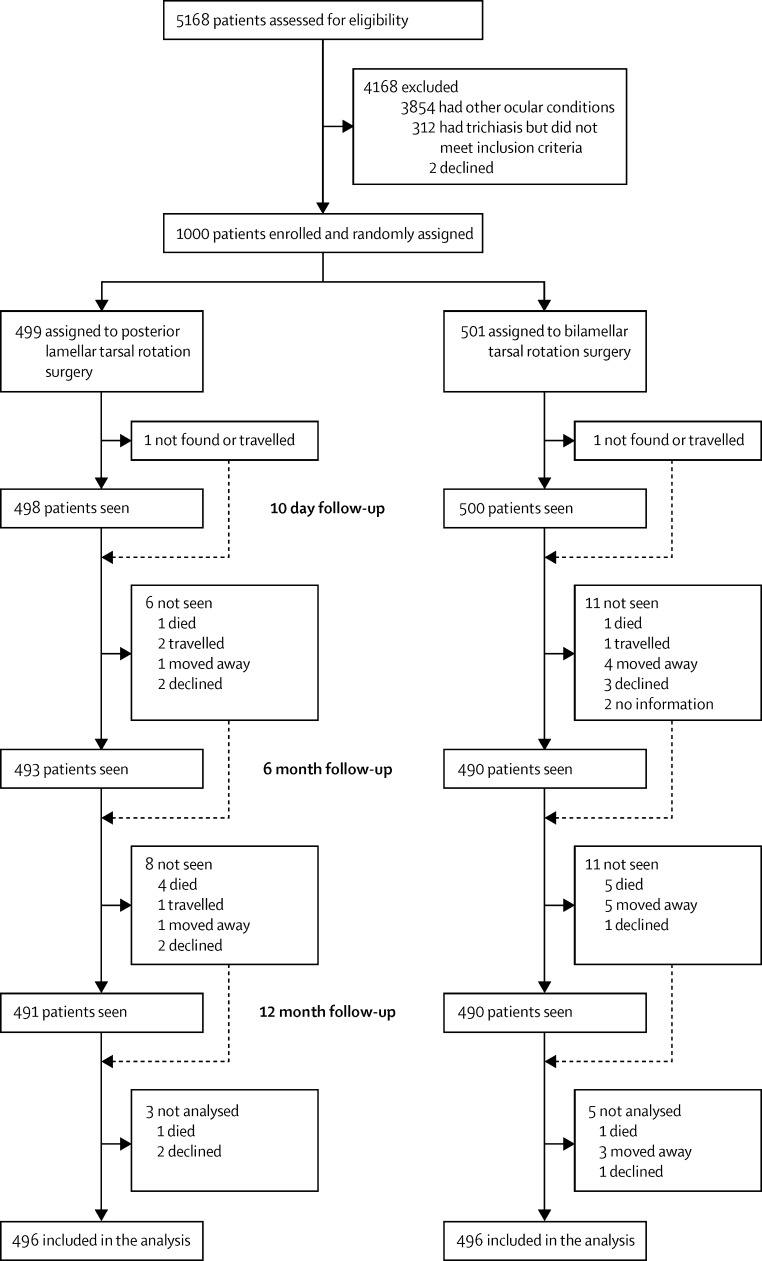
Trial profile

**Table 1:** Baseline and 12 month characteristics of participants

		**Baseline**		**12 months**	
		PLTR group (n=499)	BLTR group (n=501)	PLTR group (n=491)	BLTR group (n=490)
Sex (female)		388 (78%)	377 (75%)	..	..
Age, years (mean, SD)		47·2 (15·0)	47·5 (14·9)	..	..
Illiterate		441 (88%)	445 (89%)	..	..
Best corrected logMAR visual acuity in study eye					
	−0·1 to 0·3	141 (28%)	137 (27%)	175 (36%)	169 (34%)
	0·3 to 0·7	190 (38%)	209 (42%)	186 (38%)	212 (43%)
	0·7 to 1·1	107 (21%)	103 (21%)	90 (18%)	78 (16%)
	1·1 to 2·0	18 (4%)	18 (4%)	10 (2%)	10 (2%)
	CF/HM/PL	37 (7%)	27 (5%)	25 (5%)	15 (3%)
	NPL	6 (1%)	7 (1%)	4 (1%)	5 (1%)
	Not possible to measure	..	..	1 (<1%)	1 (<1%)
Entropion grade					
	0	11 (2%)	7 (1%)	467 (95%)	446 (91%)
	1	93 (19%)	85 (17%)	17 (3%)	39 (8%)
	2	315 (63%)	334 (67%)	6 (1%)	5 (1%)
	3	71 (14%)	66 (13%)	1 (<1%)	0
	4	9 (2%)	9 (2%)	0	0
Trichiasis (number of lashes)					
	No trichiasis	..	..	445 (91%)	405 (83%)
	None (epilating)	38 (8%)	44 (9%)	7 (1%)	13 (3%)
	1–5	316 (63%)	312 (62%)	37 (8%)	66 (13%)
	6–9	87 (17%)	87 (17%)	1 (<1%)	5 (1%)
	10–19	41 (8%)	46 (9%)	1 (<1%)	1 (<1%)
	20+	17 (3%)	12 (2%)	0	0
	Mean (SD)*	5·6 (6·6)	5·4 (5·7)	2·7 (2·7)	2·6 (2·5)
Lash location					
	None (epilating)	38 (8%)	45 (9%)	7 (15%)	13 (15%)
	Corneal with or without peripheral	450 (90%)	451 (90%)	30 (65%)	59 (69%)
	Peripheral only	11 (2%)	5 (1%)	9 (20%)	13 (15%)
Corneal opacity					
	None (CC0)	121 (24%)	132 (26%)	155 (32%)	159 (32·5)
	Peripheral (CC1)	204 (41%)	201 (40%)	140 (29%)	157 (32·0)
	Off centre faint (CC2a)	94 (19%)	94 (19%)	98 (20%)	85 (17%)
	Off centre dense (CC2b)	19 (4%)	11 (2%)	7 (1%)	4 (1%)
	Central faint (CC2c)	48 (10%)	50 (10%)	77 (16%)	76 (16%)
	Central dense (CC2d)	7 (1%)	7 (1%)	10 (2%)	5 (1%)
	Total central dense (CC3)	4 (1%)	6 (1%)	2 (<1%)	4 (1%)
	Phthisis (CC4)	2 (<1%)	0	2 (<1%)	0
Tarsal conjunctiva inflammation					
	None (P0)	6 (1%)	9 (2%)	9 (2%)	12 (2%)
	Mild (P1)	117 (23%)	131 (26%)	104 (21%)	98 (20%)
	Moderate (P2)	306 (61%)	297 (59%)	332 (68%)	321 (66%)
	Severe (P3)	70 (14%)	64 (13%)	46 (9%)	59 (12%)
Tarsal conjunctival scarring					
	None (C0)	0	0	..	..
	Mild (C1)	51 (10%)	56 (11%)	..	..
	Moderate (C2)	373 (75%)	367 (73%)	..	..
	Severe (C3)	75 (15%)	78 (16%)	..	..
Recurrent trichiasis by surgeon†					
	1	..	..	8/89 (9%)	27/91 (30%)
	2	..	..	14/95 (15%)	17/93 (18%)
	3	..	..	12/84 (14%)	17/85 (20%)
	4	..	..	10/92 (11%)	17/91 (19%)
	5	..	..	6/47 (13%)	12/47 (26%)
	6	..	..	13/89 (15%)	20/89 (22%)

Data are n (%) unless otherwise stated. BLTR=bilamellar tarsal rotation. PLTR=posterior lamellar tarsal rotation. CF=counting fingers. HM=hand movement. PL=perception of light. NPL=no perception of light.

* Excluding those with no lashes touching the eyeball.

† Data are n/N (%).

**Table 2:** Secondary clinical outcomes and changes in clinical phenotype at 12 months

		**PLTR group**	**BLTR group**	**OR or RRR (95% CI)**	**p value**
Cumulative recurrence by baseline trichiasis severity*					
	Minor trachomatous trichiasis	26/266 (10%)	36/257 (14%)	1·47 (0·85–2·53)	0·16
	Major trachomatous trichiasis	37/230 (16%)	74/239 (31%)	2·29 (1·46–3·59)	0·0003
Cumulative recurrence by baseline entropion severity*					
	None or mild	11/102 (11%)	28/90 (31%)	3·98 (1·80–8·80)	0·0007
	Moderate	37/314 (12%)	59/331 (18%)	1·59 (1·02–2·49)	0·04
	Severe	15/80 (19%)	23/75 (31%)	2·04 (0·95–4·37)	0·07
Number of recurrent lashes (mean, SD)†		2·67 (2·72)	2·65 (2·45)	0·97‡ (0·71–1·32)	0·84
Types of recurrent lashes§					
	Entropic	4/46 (9%)	10/85 (12%)	1·79¶ (0·48–6·69)	0·38
	Metaplastic (base outcome)	32/46 (70%)	55/85 (65%)	1	..
	Misdirected	3/46 (6%)	7/85 (8%)	1·37¶ (0·32–5·99)	0·67
	Epilating	7/46 (15%)	13/85 (15%)	1·26¶ (0·43–3·69)	0·68
Location of recurrent lashes§					
	Corneal or corneal and peripheral (base outcome)	30/46 (65%)	59/85 (69%)	1	..
	Peripheral	9/46 (20%)	13/85 (15%)	0·82¶ (0·30–2·22)	0·69
	Epilating	7/46 (15%)	13/85 (15%)	1·08¶ (0·37–3·12)	0·89
Visual acuity change||					
	Worse	123/490 (25%)	111/489 (23%)	0·97 (0·77–1·23)	0·81
	Same	172/490 (35%)	198/489 (41%)		
	Better	195/490 (40%)	180/489 (37%)		
Contrast sensitivity||					
	Worse	114/490 (23%)	100/489 (20%)	1·05 (0·83–1·32)	0·71
	Same	149/490 (30%)	165/489 (34%)		
	Better	227/490 (46%)	224/489 (46%)		
Corneal opacity change||					
	More opacity	84/491 (17%)	68/490 (14%)	1·19 (0·92–1·55)	0·19
	No change	329/491 (67%)	338/490 (69%)		
	Less opacity	78/491 (16%)	84/490 (17%)		
Entropion grade change||					
	<2 grade change or no change	119/491 (24%)	113/490 (23%)	1·07 (0·79–1·44)	0·67
	≥2 grade change	372/491 (76%)	377/490 (77%)		

Data are n/N (%), unless otherwise stated. BLTR=bilamellar tarsal rotation. PLTR=posterior lamellar tarsal rotation. OR=odds ratio. RRR=relative risk ratio.

* Analysis done using logistic regression adjusted for surgeon to see the effect of the two surgical procedures on cumulative recurrence (by 12 months) across baseline trichiasis and entropion severity level.

† Analysis done using negative binomial regression.

‡ Incidence rate ratio.

§ Multinomial logistic regression.

¶ Relative risk ratio.

|| Ordinal logistic regression.

**Table 3:** Complications and eyelid contour abnormalities

		**PLTR group**	**BLTR group**	**OR or RRR (95% CI)**	**p value**
Intraoperative or postoperative bleeding*					
	Mild	490/499 (98%)	477/501 (95%)	2·76 (1·27–6·00)	0·01
	Moderate	8/499 (2%)	18/501 (4%)		
	Excessive	1/499 (<1%)	6/501 (1%)		
Sign of infection at 7–14 days†‡		9/498 (2%)	37/500 (7%)	4·44 (2·11–9·33)	0·0001
Granuloma by 12 months†		26/496 (5%)	11/496 (2%)	0·41 (0·20–0·83)	0·01
Lagophthalmos (present)		3/491 (1%)	7/490 (1%)		
Eyelid contour abnormality at 12 months§					
	None (base outcome)	371/491 (76%)	404/490 (82%)	1	..
	Clinically non-significant (mild)	89/491 (18%)	49/490 (10%)	0·50¶ (0·34–0·73)	0·000
	Clinically significant (moderate-to- severe)	31/491 (6%)	37/490 (8%)	1·10¶ (0·66–1·81)	0·72
Central correction at 12 months§					
	Corrected (base outcome)	468/491 (95%)	454/490 (93%)	1	..
	Over-corrected	12/491 (2%)	6/490 (1%)	0·52¶ (0·19–1·39)	0·19
	Under-corrected	11/491 (2%)	30/490 (6%)	2·81¶ (1·39–5·68)	0·004
Medial correction at 12 months§					
	Corrected (base outcome)	469/491 (96%)	450/490 (92%)	1	..
	Over-corrected	0	0		..
	Under-corrected	22/491 (4%)	40/490 (8%)	1·90¶ (1·11–3·26)	0·02
Lateral correction at 12 months§					
	Corrected (base outcome)	486/491 (99%)	469/490 (96%)	1	..
	Over-corrected	1/491 (<1%)	0	..	..
	Under-corrected	4/491 (1%)	21/490 (4%)	5·44¶ (1·85–16·00)	0·002

Data are n/N (%), unless stated otherwise. BLTR=bilamellar tarsal rotation. PLTR=posterior lamellar tarsal rotation. OR=odds ratio. RRR=relative risk ratio.

* Ordinal logistic regression.

† Logistic regression.

‡ Erythematous swelling and discharge.

§ Multinomial logistic regression.

¶ Relative risk ratio.

**Table 4:** Patient-reported outcomes

		**PLTR group**	**BLTR group**	OR*(95% CI)	**p value**
Pain during surgery					
	None	441/499 (88%)	441/501 (88%)	1·04 (0·71–1·53)	0·84
	Mild	40/499 (8%)	39/501 (8%)		
	Moderate	7/499 (1%)	11/501 (2%)		
	Severe	11/499 (2%)	10/501 (2%)		
Pain between surgery and suture removal					
	None	347/498 (70%)	309/500 (62%)	1·46 (1·12–1·89)	0·004
	Mild	94/498 (19%)	107/500 (21%)		
	Moderate	38/498 (8%)	56/500 (11%)		
	Severe	19/498 (4%)	28/500 (6%)		
Satisfaction with the effect of surgery on the trichiasis at 12 months					
	Satisfied	463/491 (94%)	452/490 (92%)	1·39 (0·84–2·31)	0·20
	Neither satisfied nor dissatisfied	13/491 (3%)	16/490 (3%)		
	Dissatisfied	15/491 (3%)	22/490 (4%)		
Satisfaction with the cosmetic appearance at 12 months					
	Satisfied	465/491 (95%)	461/490 (94%)	1·14 (0·66–1·97)	0·64
	Neither satisfied nor dissatisfied	10/491 (2%)	7/490 (1%)		
	Dissatisfied	16/491 (3%)	22/490 (4%)		

Data are n/N (%), unless stated otherwise. BLTR=bilamellar tarsal rotation. PLTR=posterior lamellar tarsal rotation. OR=odds ratio.

* Ordinal logistic regression.
